# AlgaGEM – a genome-scale metabolic reconstruction of algae based on the *Chlamydomonas reinhardtii* genome

**DOI:** 10.1186/1471-2164-12-S4-S5

**Published:** 2011-12-22

**Authors:** Cristiana Gomes de Oliveira Dal’Molin, Lake-Ee Quek, Robin W Palfreyman, Lars K Nielsen

**Affiliations:** 1 Australian Institute for Bioengineering and Nanotechnology, The University of Queensland, Brisbane Qld 4072 Australia

## Abstract

**Background:**

Microalgae have the potential to deliver biofuels without the associated competition for land resources. In order to realise the rates and titres necessary for commercial production, however, system-level metabolic engineering will be required. Genome scale metabolic reconstructions have revolutionized microbial metabolic engineering and are used routinely for *in silico* analysis and design. While genome scale metabolic reconstructions have been developed for many prokaryotes and model eukaryotes, the application to less well characterized eukaryotes such as algae is challenging not at least due to a lack of compartmentalization data.

**Results:**

We have developed a genome-scale metabolic network model (named AlgaGEM) covering the metabolism for a compartmentalized algae cell based on the *Chlamydomonas reinhardtii* genome. AlgaGEM is a comprehensive literature-based genome scale metabolic reconstruction that accounts for the functions of 866 unique ORFs, 1862 metabolites, 2249 gene-enzyme-reaction-association entries, and 1725 unique reactions. The reconstruction was compartmentalized into the cytoplasm, mitochondrion, plastid and microbody using available data for algae complemented with compartmentalisation data for *Arabidopsis thaliana*. AlgaGEM describes a functional primary metabolism of *Chlamydomonas* and significantly predicts distinct algal behaviours such as the catabolism or secretion rather than recycling of phosphoglycolate in photorespiration. AlgaGEM was validated through the simulation of growth and algae metabolic functions inferred from literature. Using efficient resource utilisation as the optimality criterion, AlgaGEM predicted observed metabolic effects under autotrophic, heterotrophic and mixotrophic conditions. AlgaGEM predicts increased hydrogen production when cyclic electron flow is disrupted as seen in a high producing mutant derived from mutational studies. The model also predicted the physiological pathway for H*_2_* production and identified new targets to further improve H_2_ yield.

**Conclusions:**

AlgaGEM is a viable and comprehensive framework for *in silico* functional analysis and can be used to derive new, non-trivial hypotheses for exploring this metabolically versatile organism. Flux balance analysis can be used to identify bottlenecks and new targets to metabolically engineer microalgae for production of biofuels.

## Background

Microalgae are receiving increased attention as the search for sustainable and profitable biofuel feedstocks progresses. Algae-derived hydrogen, methane, triacylglycerols, and ethanol could all serve as potential biofuels [1-3], but many challenges remain to be addressed [4,5]. In order to realise the rates and titres necessary for commercial production, system-level metabolic engineering will be required [6].

In modern, system-level microbial metabolic engineering, genome scale metabolic reconstructions (GEMs) are used to integrate and analyse large ‘omics datasets as well as to evaluate designs *in silico*. A GEM maps annotated metabolic genes and proteins to reactions based on the current best understanding of a given organism. A growing number of metabolic engineering studies have demonstrated the use of well-curated GEMs to generate strain designs that are neither intuitive nor obvious [7-12].

Currently there is no genome scale reconstruction available for algae. The first attempt to reconstruct a large metabolic reconstruction of algae (based on *Chlamydomonas reinhardtii*) featured 484 reactions and 458 metabolites located in the chloroplast, cytosol and mitochondria [13] . An independent model featured 259 reactions and 267 metabolites localized to the cytosol, mitochondria, chloroplast, glyoxysome, and flagellum [14]. Despite the importance of these models, curation of cellular compartmentalization and genomic information was limited to central metabolism. Furthermore, in their current format, such models do not allow for the integration of other omics data (proteome, transcriptome and metabolome) for a system-level assessment of *C. reinhardtii*. For this, a full GEM is required.

GEMs have been developed for several model eukaryotes: yeast [15], mouse [16], human [17] and Arabidopsis [18]. For algae and other less extensively studied eukaryotes, a major challenge is the scarcity of data regarding compartmentalisation. An approach to overcome this shortfall in information is to use the compartmentalisation data for related organisms (here Arabidopsis), where no biochemical data for algae exists. We recently used this approach for the metabolic reconstruction of GEMs for the C4 grasses, maize, sorghum and sugarcane, and the resultant model was able to predict differential protein expression between mesophyll and bundle sheath, a unique C4 phenomenon [19]. The metabolism of single-cellular *C. reinhardtii*, however, has several features distinct from plants, including the presence of fermentative pathways, an inability to utilize sugars and a distinct mechanism for photorespiration.

In this paper, we develop the first compartmentalized, genome-scale model of algae metabolism (named AlgaGEM) based on the *C. reinhardtii* genome and a comprehensive evaluation of biochemical evidence found in literature complemented with missing compartmentalisation data derived from the GEM for Arabidopsis, AraGEM [18]. AlgaGEM captures the unique algae phenotypes, identifies pathways known to be important during anaerobic growth and accurately predicts the effect of a known mutation on hydrogen production. The success highlights the potential of using chimeric models to access the immensely powerful tools available for analysing GEMs, when working with biochemically less characterized eukaryotes.

## Methods

### Genome-scale metabolic reconstruction and functional annotation

**Figure 1:**
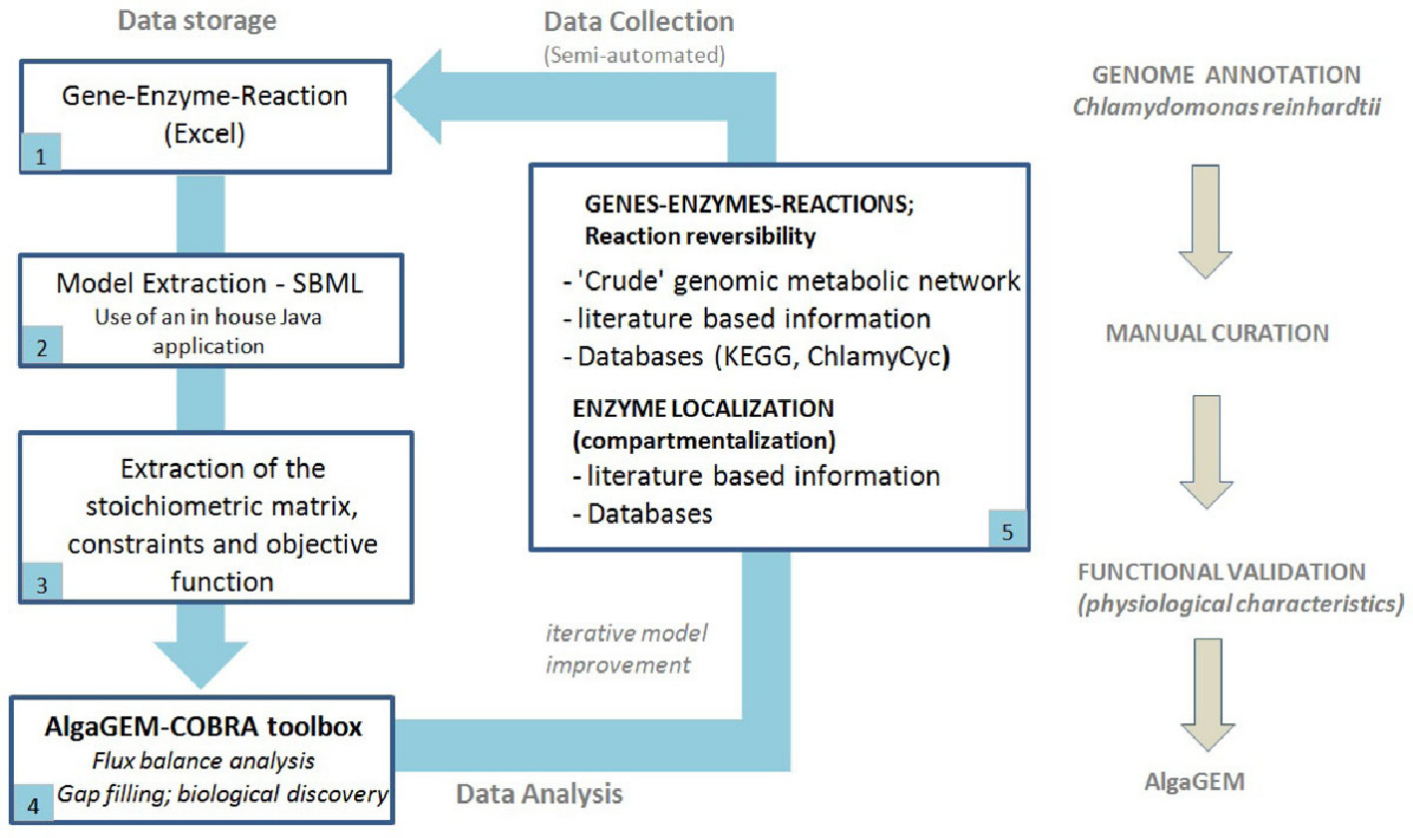
**The process for genome-scale model reconstruction.** (1) Metabolic properties including associations between genes, enzymes and reactions were extracted from the genomic metabolic databases, stored and curated in an Excel spread-sheet; (2) a 2D reaction centric SBML representation was generated using an in house java application. (3) The stoichiometric matrix, as well as reversibility constraints and the objective function were extracted from the SBML; (4) the relevant linear programming problems were solved using the COBRA toolbox [37]. (5) The model was refined in an iterative process, accessing the best available information in the literature and online data sources to achieve metabolic functionality.

The genome-scale reconstruction process was adapted from the method applied to the GEM of *Mus musculus *[20], *Arabidopsis* (AraGEM) [18], maize sorghum and sugarcane (C4GEM)[19] (Figure 1). The core of the algae genome-scale model (AlgaGEM ver 1.0) was reconstructed from the *C. reinhardtii* gene and reaction database publicly available from Kyoto Encyclopedia of Genes and Genomes (KEGG) (Release 54.1, May 1, 2010) [21]. The reconstruction retained all reaction attributes from KEGG, including unique reactions, compound IDs and reaction reversibilities. In a few cases, KEGG uses multiple labels to describe the same compound, e.g., the use of non-specific and specific references to sugar stereoisomers (e.g., D-Glucose versus α-D-Glucose). Each such multiplicity was resolved as described previously [18,20]. Another KEGG related issue addressed is the presence of unbalanced reactions, typically for (i) the synthesis or breakdown of polymers (e.g., DNA + nucleotide = DNA), (ii) use of generic groups “R” and (iii) the consumption or production of H_2_O, H^+^, and redox equivalents (e.g., NAD(P)H). In AlgaGEM, polymers are described in the form of their corresponding monomers and the use of the generic atom “R” was avoided.

In order to capture metabolism accurately, AlgaGEM was compartmentalised into cytosol, mitochondrion, plastid and microbody (functionally equivalent to peroxisomes in plants). The available metabolic pathways databases like KEGG [22] and BioCyc [23] do not capture the compartmentalization of metabolism in eukaryotes and we first attempted to use a protein subcellular localization predictive tool (WPsort, http://wolfpsort.org/)[24]. The predictions, however, were ambiguous or inconsistent across the organelles and did not produce a biochemically functional network. Instead, compartmentalisation was performed based on (a) experimental evidence whenever available from the literature [25-36] (see additional file 1; Table S1) and available on line resources (Table 1) or (b) localisation of enzyme homologs in Arabidopsis (AraGEM) [18]. Enzymes with no experimental data and no homolog found in Arabidopsis (e.g., fermentative reactions) were assigned to the cytosol per default. Transport reactions between cytoplasm and extracellular space and between cytoplasm and organelles are poorly annotated and were added manually based on transport reactions reported in the literature.

**Table 1:** Online resources for the reconstruction of the metabolic network of *Chlamydomonas reinhardtii*

Database	Link
***Genome Database ***	
DOE Joint Genome Institute (JGI); *Chlamydomonas reinhardtii* v4.0	http://genome.jgi-psf.org/Chlre4/Chlre4.home.html
An Online Informatics Resource for *Chlamydomonas* (*Chlamy Center*)	http://www.chlamy.org/chlamydb.html

***Pathway Databases ***	
Kyoto Encyclopedia of Genes and Genomes (KEGG)	http://www.genome.jp/kegg/pathway.html
ChlamyCyc	http://chlamyto.mpimp-golm.mpg.de/chlamycyc/index.jsp
Metacyc	http://metacyc.org/
ExPASy Biochemical Pathways	http://www.expasy.ch/cgi-bin/search-biochem-index

***Enzymes Databases***	
ExPASy Enzyme Database	http://ca.expasy.org/enzyme/
	http://www.brenda-enzymes.info/

Enzyme/Protein Localization and others Databases*	
AraPerox (Arabidopsis Protein from Plant Peroxisomes)	http://www.araperox.uni-goettingen.de/
SUBA (Arabidopsis subcellular database)	http://www.plantenergy.uwa.edu.au/applications/suba2/index.php
PPDB (Plant proteome database)	http://ppdb.tc.cornell.edu/default.aspx
UniproKB/SwissProt	http://ca.expasy.org/sprot/relnotes/relstat.html
Transport DB	http://www.membranetransport.org/

*Manual curation based on literature and homology sequence.

AlgaGEM was compiled and curated in Excel (Microsoft Corporation) for ease of annotation and commenting (Figure 1). From this gene centric database, a 2D reaction centric SBML (System Biology Markup Language, http://www.sbml.org) representation was generated using an in house Java (Oracle Corporation) application. As there is currently no specific element in SBML allocated to store the gene–protein–reaction associations (e.g. splice-variants, isozymes, protein complexes), these were added as notes to the reaction elements. Constraint-based reconstruction and analysis was performed using COBRA toolbox (http://opencobra.sourceforge.net/) [37]; a set of MATLAB scripts for constraint-based modelling run from within the MATLAB environment (Version 7.3, The MathWorks, Inc.). Simulated flux distributions were visualized on a metabolic flux map (for the visualization of overall changes in the central metabolism of a compartmentalized algal cell) drawn in Excel.

AlgaGEM was evaluated for its ability to produce major biomass components and cofactors (Table 2) under autotrophic (photons as energy source, CO_2_ as carbon source and nitrate or ammonia as nitrogen source), heterotrophic (acetate as carbon source, nitrate and/or ammonia and/or amino acids as nitrogen sources) and mixotrophic (photons as energy source, CO_2_ and acetate as carbon sources and nitrate or ammonia as nitrogen source) conditions. For each biomass component in Table 2 and each growth condition, the following linear programming problem was formulated and solved

**Table 2:** List of biomass components

Carbohydrates and sugars	Starch, sucrose, fructose, glucose, maltose
	
Protein (amino acids)	Alanine, arginine, aspartate, asparagine, cystein, lysine, leucine, isoleucine, glutamate, glutamine, histidine, methionine, phenylalanine, proline, serine, tyrosine, tryptophan, valine
Nucleotides	ATP, GTP, CTP, UTP, dATP, dGTP, dCTP, dTTP
Fatty acids	C16:0 (Palmitic acid)
Vitamins and cofactors	Biotin, coenzyme A, riboflavin, folate, chlorophyll, nicotinamide, thiamine, ubiquinone,

where *v*_i_ is the corresponding biomass drain reaction. Where the maximum production rate of a biomass component was zero, gap analysis was performed. Some gaps were readily filled based on inspection of the corresponding pathways in KEGG, ChlamyCyc [38] and other available databases (Table 1). Others, such as inconsistent irreversibility constraints, stoichiometry errors, compound names, compartmentalization errors or missing transporters, required sequential tracing through the model to identify breakpoints and careful evaluation of the possible causes.

Once network gaps were closed, the individual biomass accumulation terms were combined into an overall biomass synthesis equation, with the appropriate coefficients assigned to each precursor to define the composition of biomass. The overall biomass synthesis equation depends on growth conditions and was designed to represent autotrophic, heterotrophic and mixotrophic conditions based on literature data [13]. Trace elements were not included in the biomass equation, since their contribution to overall flux is trivial.

### Model simulations

After curation, AlgaGEM was used as a framework to test cell optimality and maximum bioproduct performance under different conditions. Constraints were applied and flux balance analysis was performed in MATLAB using COBRA Toolbox [37]. The COBRA files can be downloaded from the additional files (see additional file 2; AlgaGEM-COBRA). The folders include the SBML file and the respective constraints to represent each metabolic scenario. The minimum set of constraints imposed to represent the different growth conditions are shown in Table 3.

**Table 3:** Minimal set of constraints imposed to represent different growth condition

Inputs, outputs and constraints	Autotrophic	Heterotrophic	Mixotrophic
C source: CO_2_ uptake	+	-	+
C source: Acetate uptake	-	+	+
Photons uptake (free flux)	+	-	+
Optimization 1: minimize uptake of	Photons	Acetate	Photons
Optimization 2: maximize product	H_2_	H_2_	H_2_
Biomass rate (fixed) *	0.059 h^-1^	0.035 h^-1^	0.066 h^-1^

*Biomass rate and biomass equation was used for each growth regime, based on measurements found in the literature [13].

#### Minimize energy and carbon source

The final model was evaluated through the estimation of the flux distributions in three growth conditions: autotrophic, heterotrophic and mixotrophic. The flux distributions were determined using linear programming

i.e., the distributions that minimize the use of the key energy substrate (photons or acetate), while achieving a specified growth rate.

#### Maximize bio-product: H_2_

We also used the model to test the network capacity to maximize H_2_ under different growth conditions.

i.e., the flux distributions that maximize H_2_ synthesis, while achieving a specified growth rate under autotrophic, mixotrophic or heterotrophic condition.

## Results and discussion

### Characteristics of the reconstructed network

Genome-scale metabolic models bridge the gap between genome-derived biochemical information and metabolic phenotypes in a structured manner. The genome-scale reconstruction (AlgaGEM, Ver 1.0) contains 2249 gene-reaction association entries, 1725 unique reactions and 1869 metabolites distributed across 4 cellular compartments (Table 4). The active scope of the AlgaGEM includes glycolysis (plastidic and cytosolic), the pentose phosphate pathway (PPP) (plastidic and cytosolic), tricarboxylic acid cycle (TCA cycle), light and dark reactions (Calvin cycle), fatty acid synthesis, beta-oxidation, glyoxylate cycle, photorespiratory cycle and fermentative reactions. The current version of AlgaGEM has not been tested for its coverage of secondary metabolism and some alternative fermentative pathways that are not well understood or described at the subcellular level for algae.

**Table 4:** Characteristics of the reconstructed genome-scale model (AlgaGEM)

Metabolic properties	Total
Gene-reaction-association entries	2249
Unique metabolic reactions	1725
Unique ORFs	866
Metabolites	1862
Cellular compartments	4
Biomass drains	39
Intercellular transporters	24
Inter-organelle transporters	79
Gaps (non-enzymatic reactions)	3

Forty two (42) biomass drain equations describe the accumulation of carbohydrates, sugars, amino acids and fatty acids, representing the major biomass drains for an algal cell (Table 2). At present, fatty acid biosynthesis is limited to palmitic acid biosynthesis in chloroplasts. The biosynthetic pathways of a limited number of vitamins and co-factors have been curated to date. Twenty-four (24) intercellular exchange reactions (cytoplasm–extracellular) have been included to describe the uptake of light (absorbed photons), and the uptake/secretion of inorganic compounds (CO_2_, H_2_O, HCO_3_-; O_2_, NO_3_, NH_3_, H_2_S, SO_4_^2–^, PO_4_^3–^), translocation of fermentative products (like acetate, glycolate, lactate and ethanol), H_2_ and amino acids (glutamine, glutamate, aspartate, alanine and serine). Together with biomass drains (39), the intercellular exchangers define the broad physiological domain of the model, that is, the curated aspects of *C. reinhardtii* primary metabolism captured by AlgaGEM. Inter-organelle transporters were added based on the biochemistry information available for algae (see additional file 1; Table S1). When not available, we used information that supports inter-organelle transporters for Arabidopsis (E.g.; Transport DB, Table 1). A total of 79 inter-organelle transporters were introduced in the model to achieve metabolic functionality. Apart from nomenclature and cellular compartmentalization issues, only three additional reactions without gene associations (non-enzymatic steps) were added during model curation before the model was able to simulate growth *in silico*.

### AlgaGEM predicts phosphoglycolate catabolism in algae

The reconstruction of metabolic models for eukaryotes is challenging due to the scarcity of biochemical information at the subcellular level required for cellular compartmentalisation. AlgaGEM covers our current understanding of metabolic functionality and connectivity through different organelles for *C. reinhardtii*. It does rely, however, on AraGEM [18] for the compartmentalisation of reactions for which no data exist for *C. reinhardtii*. Given that there is a substantial, natural overlap between AlgaGEM and AraGEM with approximately 85% of all reactions present in both models, it is important to establish that AlgaGEM indeed predicts algae behaviour rather than slightly modified Arabidopsis behaviour.

Heterotrophic growth in AlgaGEM differs from AraGEM in that the former can metabolize acetate and glycolate, but lacks glucose and sucrose transporters and is unable to utilize these carbon sources from the media [33]. Moreover, AlgaGEM has fermentative reactions and produces a range of fermentative products (like H_2_, glycolate, acetate, formate, lactate, etc). These differences are the direct result of added reactions and transporters.

A more interesting difference is the way algae and plants handle the RuBisCO oxygenation reaction. Assuming that plants have evolved to optimise photon efficiency, AraGEM accurately predicts that phosphoglycolyate is recycled using the classical photorespiration cycle involving reactions in plastids, peroxisomes and mitochondria [18]. Moreover, it accurately predicts that photon requirements increase by 30-40%, if the ratio of oxygenation-to-carboxylation is 1:4.

Photorespiration of *C. reinhardtii* deviates from the classical plant photorespiration in that instead of oxidizing glycolate to glyoxylate via glycolate oxidase in peroxisomes, *C. Reinhardtii* and many other microalgae utilize glycolate in mitochondria [25,39,40]. In addition, because molecular O_2_ is not an electron acceptor for glycolate dehydrogenase, glycolate oxidation catalysed by this enzyme does not produce H_2_O_2_, so catalase should not be required for the photorespiration cycle in algae. Instead, glycolate dehydrogenase is expected to contribute electrons to the mitochondrial respiratory electron transport through reduction of ubiquinone pool [40]. AlgaGEM accurately predicts that algae will catabolise rather than recycle phosphoglycolate, if sufficient oxygen is available and energy is needed, or alternatively secrete glycolate to the environment, as has also been observed [41,42].

### AlgaGEM predicts the physiological pathways used for H_2_ production in *Chlamydomonas* under dark condition

**Figure 2:**
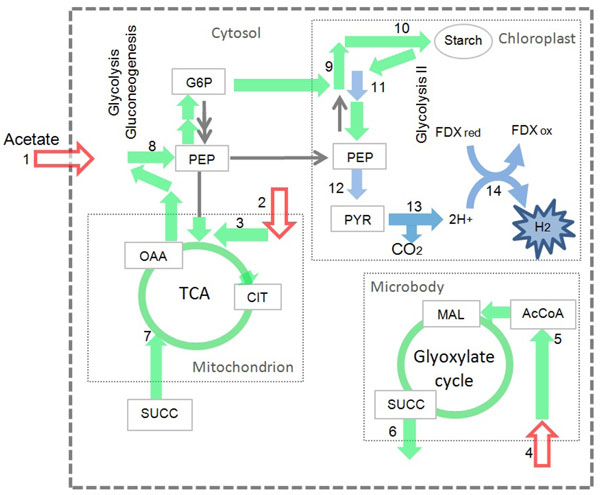
**Increased fluxes through the physiological pathways used for H_2_ production in *Chlamydomonas* under dark condition.** Acetate is assimilated (red arrows) and starch is produced through gluconeogenic conversion. Green arrows highlight increased fluxes through acetate metabolism, starch synthesis/degradation and the physiological pathways for H_2_ production in Chlamydomonas. The blue arrows are the increased fluxes through enzymes involved in H_2_ synthesis. Numbers represent the main enzymatic and transport reactions: (1) acetate assimilation; (2,4) acetate transporter; (3,5) acetyl-Coa synthetase; (6,7) succinate transporter; (8) phosphoenolpyruvate carboxykinase; (9) phosphoglucomutase (10) 1,4-alpha-glucan branching enzyme; (11) glyceraldehyde 3-phosphate dehydrogenase; (12) pyruvate kinase; (13) pyruvate ferredoxin oxidoreductase (PFR1); (14) ferredoxin hydrogenase. GLU: glucose; PEP: Phosphoenolpyruvate; G6P: glucose 6-phosphate; SUCC: Succinatate; MAL: malate; CIT: citrate; OAA: oxaloacetate; FDX red: reduced ferredoxin; FDX ox: oxidized ferredoxin.

**Figure 3:**
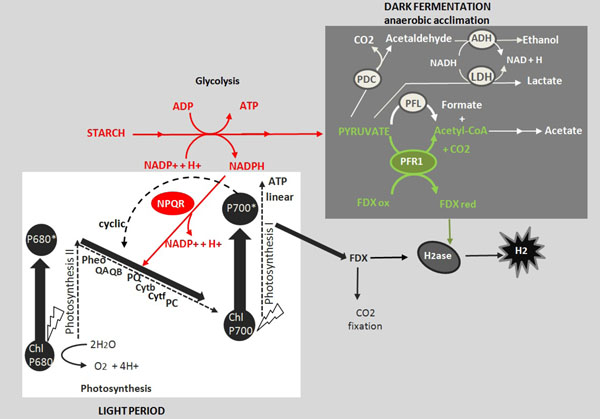
**Simplified illustration of the physiological pathways used for H_2_ production in *Chlamydomonas* (Adapted from The Chlamydomonas Sourcebook **[44]**).** The two photoproduction pathways involving PSII and PSI under the light period are showed in black. Electrons excited to higher energy (low potential) by PSI are able to reduce ferredoxin (FDX), the physiological electron donor to hydrogenase. Both the PSII-dependent and NPQR-dependent (red) pathways require reduction of the PQ pool and PSI activity. In the case of the PSII-independent pathway (reactions in red), reducing power formed by the catabolism of organic substrates is used for reduction of the PQ pool. During dark fermentation the oxidation of pyruvate catalysed by PFR (green reactions) is used to reduce ferredoxin and putatively mediates the observed production of H_2_ in the dark. White reactions show the parallel main fermentative products from pyruvate, competing with H_2_. Dashed arrows show linear and cyclic electron flow.

Although substantial insights regarding algal H_2_ production exist [1,43], critical aspects regarding the hydrogenase activity remain unresolved and new advances are required to define more clearly the metabolic and enzymatic processes influencing algal H_2_ production. AlgaGEM was used to capture contrasts in the metabolism when H_2_ is maximized during heterotrophic conditions (dark). Firstly, flux distribution was calculated by linear programming assuming carbon efficiency and minimum acetate usage to maintain cell growth under dark condition (see optimality criterion in methods). Secondly, the calculated optimum uptake rate of acetate to maintain biomass growth under heterotrophic conditions was fixed and H_2_ production was maximized to find the main metabolic changes. Ultimately we maximized H_2_ production under different uptake rates of acetate. Figure 2 highlights in green the increased fluxes (more than 20% increased) through different cell compartments when H_2_ production is maximized. During dark periods and using acetate as the sole carbon source, acetate is assimilated and storage compounds like starch are produced through gluconeogenic conversion [44]. Fluxes are increased through the glyoxylate cycle in the microbody and mitochondrial reactions, where acetate is mainly metabolised (showed by red arrows). It is believed that acetate is primarily converted to acetyl CoA via the glyoxylate cycle [44]. The glyoxylate cycle can generate one molecule of succinate as a net product from two molecules of acetate. As shown in Figure 2, the model predicts that acetate assimilation proceeds along the same pathway as gluconeogenic fatty acid conversion in oil seed. Succinate produced in the glyoxylate cycle is transported from the microbody and converted to malate and oxaloacetate in the mitochondrion. Oxaloacetate is decarboxilated to phosphoenolpyruvate in the cytosol, and phosphoenolpyruvate is converted into sugars phosphates. Our simulations show increased flux through phosphoenolpyruvate transported to chloroplast, and subsequent conversion into sugars and starch. The blue arrows highlight flux increased through enzymes involved in H_2_ synthesis. These steps refers to chloroplastic glyceraldehyde 3-phosphate dehydrogenase which supply reducing power during glycolysis and subsequent oxidation of pyruvate in the chloroplast catalysed by pyruvate ferredoxin oxidoreductase (PFR1) yielding acetyl-CoA, reduced ferredoxin and CO_2_ and mediates the observed production of H_2_ in the dark [34,45-47] . These steps are also summarized in Figure 3 where red reactions represent catabolism of organic substrates (reducing power supply) and green reactions represent dark fermentation. Both of *C. reinhardtii’s* [Fe]-hydrogenases, HydA1 and HydA2, catalyze H_2_ production using electrons from ferredoxin. In several species of anaerobic microbes the decarboxylation of pyruvate to acetyl-coA by PFR1 is linked to hydrogen production via the reduction of ferredoxin [34,45-47]. The simulated flux distribution highlights the physiological pathways used for H_2_ production in *Chlamydomonas* when growing under dark conditions, as reported in the literature [44][34,45-47]. The identification in *C. reinhardtii* of a putative PFR1 gene, evidence of its coexpression with HydA1 and HydA2 [46][44] and our flux analysis suggests fermentative carbon metabolism and dark, anaerobic H_2_ production may be linked via ferredoxin. This could explain the source of electrons in the dark, fermentative H_2_ production, a discovery with the potential to improve dark H_2_ conversion efficiency. The model suggests that over expression of PFR1 gene should improve H_2_ yield under dark conditions by supplying the electron donor to hydrogenase. Further experimental investigation is required to validate this hypothesis.

### AlgaGEM predicts increased hydrogen production when cyclic electron flow is disrupted under mixotrophic conditions

*C. Reinhardtii* produces hydrogen under heterotrophic and mixotrophic conditions (Figure 3). The rates are generally low and metabolic engineering strategies are being explored to improve production rates. Mutational studies identified a high producing, state transition mutant, *Stm6*, which is blocked in state 1 photosynthesis and hence has greatly inhibited cyclic electron flow around photosystem I [48].

**Figure 4:**
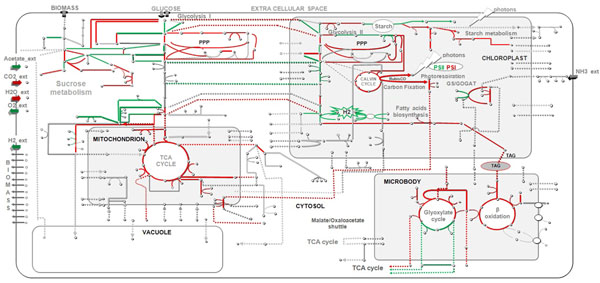
**Overall metabolic changes when H_2_ is maximized under mixotrophic condition.** Comparison of fluxes between H_2_ producer and no H_2_ producer type. Solid lines represent fluxes, dashed lines represent transporters and vertices represent the metabolites. Green and red lines highlight fluxes that have increased and decreased, respectively when H_2_ is produced. Gray lines represent fluxes that have not changed significantly.

We used AlgaGEM to simulate the metabolic changes when H_2_ is produced under mixotrophic growth. We compared flux distributions for two optimality criteria: maximization of H_2_ (hypothetical H_2_ producer) and minimum energy resource usage to maintain normal growth rate (wild type). Flux simulations are provided in detail (see additional file 3; Table S2) and illustrated in Figure 4. Key changes are summarised in Table 5. Under mixotrophic conditions and constant illumination (65μE/m^2^/s), the model predicted about an 8-fold increase in H_2_ production, if cyclic electron flow is inhibited. This is in line with observations for the *stm6* mutant, which produced 5-13 fold more H_2_ compared to the wild type [48]. Cyclic electron flow is normally used to balance requirements for redox with requirements for ATP. When cyclic electron flow is inhibited, H_2_ production becomes a release valve for excess redox.

**Table 5:** Upregulation of key target enzymes when H_2_ is produced under mixotrophic conditions, highlighted by AlgaGEM

Step reaction	Regulation (up/down)	Reference*
Cyclic electron flow (PSI)	-	[48]
Linear electron flow (PSII)	+	[48]
TCA cycle/mitochondria	-	[48]
Fe-Hydrogenases	+	[29,31,35,49-52]
Ferredoxin-NADP+ reductase (FNR)	+	[53]
Glyceraldehyde 3-P-dehydrogenase	+	[54-57]
Pyruvate ferredoxin oxireductase	+	[58-60]
Calvin Cycle/CO2 assimilation	-	[44]
Acetate assimilation	+	[44]
Gluconeogenesis (cytosolic)	+	Not found
Pentose phosphate pathway (cytosolic)	-	Not found
Pentose phosphate pathway (plastidic)	+	Not found
Beta oxidation/glyoxylate cycle	-	Not found
glutamate synthase (ferredoxin);GSGOGAT	+	Not found
glutamine synthetase; GS/GOGAT	-	Not found

*Results supported by evidences found in the literature. Not found: model highlights new targets in different pathways to improve H_2_ that need further experimental investigation for model validation.

Also in line with observations for the *Stm6* mutant, the model predicted increased linear electron flow and reduced mitochondrial TCA cycle activity (Table 5). Furthermore, the model predictions agreed with observations made in other studies regarding the correlation of hydrogen production with expression of various genes, including increased activity of reactions directly involved in hydrogen production (Fe-Hydrogenases, Ferredoxin-NADP+ reductase, glyceraldehyde -3-phosphate dehydrogenase, and pyruvate ferredoxin oxireductase) and a shift carbon assimilation through Calvin cycle/CO_2_ assimilation to acetate assimilation (Table 5). The model also suggested a number of possible further targets for investigation, including gluconeogenesis, pentose phosphate pathway, beta oxidation, glyoxylate cycle and GS/GOGAT.

## Conclusions

AlgaGEM is a curated, compartmentalized genome scale model of algal cell primary metabolism. Continued curation efforts will focus on closing gaps, especially in the secondary metabolism and alternative fermentative pathways (not well understood or described at organelle level for algae) and the resolution of gene product targeting where this is yet to be established. In its current version, the model covers the primary metabolism including some of the fermentative pathways. Importantly, while the model shares 85% of the reactions with AraGEM and while AraGEM was used to compartmentalise many genes, the model predicts distinct algal behaviours such as the catabolism or secretion rather than recycling of phosphoglycolate in photorespiration.

The use of AlgaGEM for *in silico* flux predictions illustrates the potential of using genome scale models to explore complex, compartmentalized networks and develop non-trivial hypotheses. The metabolic changes highlighted by AlgaGEM to increase H_2_ yield show agreement with evidence found in the literature and the model predicted the magnitude of change observed in a stage transition mutant, *Stm6*. Further experimental investigations are also suggested to test new targets. Such results support the potential use of this framework for algae metabolic engineering.

## Competing interests

The authors declare that they have no competing interests.

## Authors' contributions

CGO carried out the reconstruction and curation of AlgaGEM, performed *in silico* flux analysis and wrote the manuscript. LQ developed the method for the reconstruction and contributed in insightful discussions. RP worked on the data mining process and analysis. LKN conceived the study, performed analyses and revisions of the manuscript. All authors read and approve the final manuscript.

## Supplementary Material

Additional file 1 **Supplemental material file**. Table S1. List of inter-organelle transporters and enzymes’ localization based on biochemistry information. Click here for file

Additional file 2 **Supplemental material file**. AlgaGEM-COBRA tool box. The folders include AraGEM (sbml format) and constraints to run flux balance analysis for different metabolic scenarios using COBRA tool box. Click here for file

Additional file 3 **Supplemental material file**. Table S2. Metabolic flux simulations. Click here for file
