# Supplementary material for: AlgaGEM – a genome-scale metabolic reconstruction of algae based on the Chlamydomonas reinhardtii genome
Source: BMC Genomics. 2011 Dec 22;12(Suppl 4):S5. doi: 10.1186/1471-2164-12-S4-S5 (PMC3287588; doi:10.1186/1471-2164-12-S4-S5)
Supplement: Additional file 2 — Supplemental material file. AlgaGEM-COBRA tool box. The folders include AraGEM (sbml format) and constraints to run flux balance analysis for different metabolic scenarios using COBRA tool box. [file 1471-2164-12-S4-S5-S2.zip › Additional file 2_AlgaGEM_COBRA/Read me.docx]

# Folders content:

Folders 1 -6 contain the AlgaGEM sbml, the respective reaction constrains and objective functions for the different metabolic scenarios:

**Folder 1**; Heterotrophic condition (reference case)

Objective function: minimization of Acetate

**Folder 2**; Heterotrophic condition (hypothetical H_2_ producer)

Objective function: maximization of H_2_

**Folder 3**; Mixotrophic condition (reference case)

Objective function: minimization of photons

**Folder 4;** Mixotrophic condition (reference case)

Objective function: maximization of H_2_

**Folder 5**; Autotrophic condition (reference case)

Objective function: minimization of photons

**Folder 6**; Autotrophic condition (reference case)

Objective function: maximization of H_2_

# Using COBRA Toolbox to read AlgaGEM sbml

### COBRA Toolbox

COBRA Toolbox is a set of Matlab scripts for constraint-based modeling that are run from within the MATLAB environment. We have evaluated version 2.0 obtained from SourceForge: <http://opencobra.sourceforge.net/openCOBRA/Welcome.html>.

They provide everythijng needed to get started, including libSBML (version 4.2.0), the SBMLToolbox (3.1.2), and the glpk_mex interface to the GNU Linear Programming Kit and the COBRA Toolbox for Mac OS X 10.6 (64-bit), GNU/Linux Ubuntu 10.0 (64-bit), and Microsoft Windows 7 (64-bit).

It is recommended replacing the free glpk LP solver with a commercial solver. If working in an academic environment, it is currently possible to obtain a free academic license for MOSEK (<http://www.mosek.com/>).

In order to get started, you need to

1. Add the path to any commercial solver from within Matlab using addpath, e.g.

>> addpath 'c:\Program Files\mosek\6\toolbox\r2009b'

1. From the install directory of the COBRA Toolbox, run the initialisation routine

>> initCobraToolbox

This will add paths to all the Toolbox files and choose a solver. NB: if using a commercial solver, you may need to change order of solvers in the initCobraToolbox to make certain it picks the one you want.

### AlgaGEM sbml

The AlgaGEM COBRA zip file contains the files needed to simulate flux distribution during H2 production, namely the SBML file (AlgaGEM.xml) and three constraint files: objectiveFunction.txt; reactionConstraints.txt; boundarySpecies.txt.

In Matlab, go to the folder where the zip file was extracted to and load the model

Example: Folder 1 (heterotrophic condition)

>> model = readCbModel('AlgaGEM.xml')

model =

rxns: {1725x1 cell}

mets: {1869x1 cell}

S: [1869x1725 double]

rev: [1725x1 double]

lb: [1725x1 double]

ub: [1725x1 double]

c: [1725x1 double]

rules: {1725x1 cell}

genes: {1381x1 cell}

rxnGeneMat: [1725x1381 double]

grRules: {1725x1 cell}

subSystems: {1725x1 cell}

rxnNames: {1725x1 cell}

metNames: {1869x1 cell}

metFormulas: {1869x1 cell}

b: [1869x1 double]

description: 'AlgaGEM.xml'

The optimal solution (here minimum acetate required to meet specified biomass synthesis) can be determined using

>> solution = optimizeCbModel(model,'min',false,false)

solution =

f: 0.0071

x: [1725x1 double]

y: [1869x1 double]

w: []

stat: 1

solver: 'mosek'

time: 0.0911

The solution can be printed out using

>> printFluxVector(model, solution.x)

To test H_2_ production, the optimal solution is the flux distributions that maximize H_2_ synthesis, while achieving a specified growth rate under autotrophic, mixotrophic or heterotrophic condition and can be determined using

>> solution = optimizeCbModel(model,'max',false,false)
